# Temperature modulation with an esophageal heat transfer device- a pediatric swine model study

**DOI:** 10.1186/1471-2253-15-16

**Published:** 2015-02-04

**Authors:** Erik B Kulstad, Melissa Naiman, Patrick Shanley, Frank Garrett, Todd Haryu, Donald Waller, Farshid Azarafrooz, Daniel Mark Courtney

**Affiliations:** Department of Emergency Medicine, Advocate Christ Medical Center, Oak Lawn, IL 60453 USA; Department of Emergency Medicine, University of Illinois at Chicago, Chicago, IL 60612 USA; Center for Advanced Design, Research, and Exploration (CADRE), University of Illinois at Chicago, 1737 W. Polk Street, Suite B14, Chicago, IL 60612 USA; Advanced Cooling Therapy, 3440 S. Dearborn Street, #215-S, Chicago, IL 60616 USA; Garrett Technologies, 1955 Techny Road, Suite #1, Northbrook, IL 60062 USA; PreLabs, LLC, 33 Chicago Avenue, Oak Park, IL 60302 USA; Department of Comparative Medicine, Loyola University Medical Center, Maywood, IL 60153 USA; Department of Emergency Medicine, Feinberg School of Medicine, Northwestern University, 211 E. Ontario suite 200, Chicago, IL 60611 USA

## Abstract

**Background:**

An increasing number of conditions appear to benefit from control and modulation of temperature, but available techniques to control temperature often have limitations, particularly in smaller patients with high surface to mass ratios. We aimed to evaluate a new method of temperature modulation with an esophageal heat transfer device in a pediatric swine model, hypothesizing that clinically significant modulation in temperature (both increases and decreases of more than 1°C) would be possible.

**Methods:**

Three female Yorkshire swine averaging 23 kg were anesthetized with inhalational isoflurane prior to placement of the esophageal device, which was powered by a commercially available heat exchanger. Swine temperature was measured rectally and cooling and warming were performed by selecting the appropriate external heat exchanger mode. Temperature was recorded over time in order to calculate rates of temperature change. Histopathology of esophageal tissue was performed after study completion.

**Results:**

Average swine baseline temperature was 38.3°C. Swine #1 exhibited a cooling rate of 3.5°C/hr; however, passive cooling may have contributed to this rate. External warming blankets maintained thermal equilibrium in swine #2 and #3, demonstrating maximum temperature decrease of 1.7°C/hr. Warming rates averaged 0.29°C/hr. Histopathologic analysis of esophageal tissue showed no adverse effects.

**Conclusions:**

An esophageal heat transfer device successfully modulated the temperature in a pediatric swine model. This approach to temperature modulation may offer a useful new modality to control temperature in conditions warranting temperature management (such as maintenance of normothermia, induction of hypothermia, fever control, or malignant hyperthermia).

## Background

Modifying or influencing the temperature of patients has been shown to be important for a number of conditions. Temperature modulation includes reduction of body temperature below normal, maintenance of normal body temperature for the avoidance of febrile or hyperthermic states, and active warming of patients to avoid unintended reductions of body temperature. The strength of evidence for improved outcomes when temperature modulation is implemented in several clinical scenarios is such that it is now considered a standard of care, endorsed by major resuscitative, cardiovascular, neonatal, and/or surgical standards groups (including the American Heart Association, the International Liaison Committee on Resuscitation, the European Resuscitation Council, the National Institute of Child Health and Human Development, the National Institute for Health and Care Excellence, the Centers for Medicare and Medicaid Services via the Surgical Care Improvement Project, and the American Society of Anesthesiologists) [1–8]. In particular, adults who remain comatose after resuscitation from cardiac arrest, neonates suffering from hypoxic ischemic encephalopathy, and patients undergoing general surgical procedures longer than one hour in duration all have strong recommendations for temperature modulation. More broadly, temperature control has been shown either preliminarily, or potentially, to be beneficial for certain subsets of traumatic brain injury [9, 10], spinal cord injury [11–13], certain subsets of stroke [14–18], acute myocardial infarction [19–22], traumatic/hemorrhagic cardiac arrest [23], surgical operations lasting longer than one hour [24–29], hepatic encephalopathy [30–32], and sepsis/septic shock [33]. The challenge of maintaining operative normothermia during procedures on patients with high surface to mass ratios, such as pediatric patients, is significant, and many approaches are being developed to address this need [34–36].

**Figure 1:**
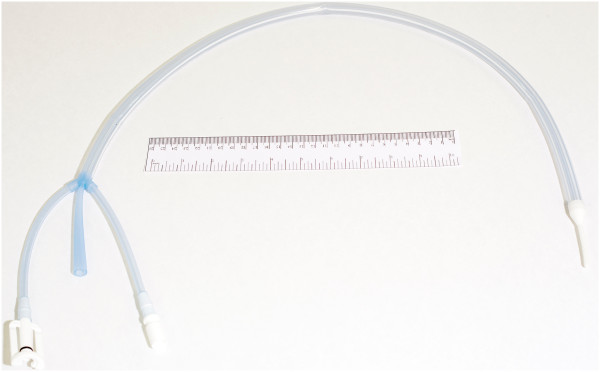
**Picture of esophageal heat transfer device.**

Leveraging the efficiencies of the heat transfer environment surrounding the esophagus, we have developed a heat transfer device that offers a new approach for modulation of patient temperature. The device is similar in size to large orogastric tubes, and incorporates internal channels to allow a closed circuit of water (serving as the heat exchange medium) provided by an external heat exchanger, with a central core serving as gastric access (Figure 1). Specifically, the device consists of a total of three lumens, one of which is isolated and serves as a channel or conduit allowing direct gastric access for suctioning and decompression, the other two of which allow parallel countercurrent flow of coolant (water) which is provided by the external chiller. Initial mathematical modeling data [37] and animal data [38] have shown good results in larger, adult-sized models; however, given the additional challenges of modulating temperature in smaller patients with larger surface to mass ratios, we sought to evaluate the ability of this device to influence temperature in a pediatric swine model, hypothesizing that clinically significant modulation in temperature (both increases and decreases) would be possible.

## Methods

### Animals

This prospective interventional study was performed by an experienced team under a protocol approved by the Institutional Animal Care and Use Committee (IACUC) of Loyola University Medical Center. The study utilized methods consistent with current veterinary and USDA standards, with a state-of-the-art, Association for Assessment and Accreditation of Laboratory Animal Care (AAALAC) International-accredited vivarium. Animal care and handling was in accord with Office of Laboratory Animal Welfare guidance for humane care and use of animals and with regulations outlined in the USDA Animal Welfare Act (9 CFR Parts 1, 2 and 3) and the conditions specified in the Guide for the Care and Use of Laboratory Animals (National Academy Press, Washington DC, 1996). Animal experimentation was required to determine the in vivo feasibility of modulating temperature with this approach, as in vitro models can only roughly approximate human cardiovascular physiology. A swine model of the size chosen has similarity in size, physiology, and thoracic anatomy to pediatric patients.

### Procedures

Three female Yorkshire swine weighing 21 kg, 22.5 kg, and 24.6 kg, for a mean 22.7 kg ± 1.8 kg, were acclimated to the facility as part of an unrelated dermal study investigating a small (2 square centimeter) region of skin for a topical pharmaceutical. Animals were given 12 hours food restriction but free access to water before the intervention. Subjects were medicated with a pre-anesthetic mix of Telazol (tiletamine/zolazepam) 4.4 mg/kg and xylazine 2.0 mg/kg intramuscularly, endotracheally intubated and anesthetized with 3% inhalational isoflurane (with concentration adjusted as needed to maintain anesthesia). No paralytics were used during any part of the study. Normal saline was instilled at a maintenance rate (2 cc/kg/hr) via ear vein. Continuous cardiac monitoring was performed with a 3-lead EKG rhythm recorder.

### Intervention

The heat exchange device was connected to an external heat exchange unit (Gaymar Medi-Therm III, Gaymar industries, Inc., Orchard Park, New York) which uses distilled water as the coolant, and the unit was powered up. The tip of the device was lubricated with a water soluble lubricant and inserted through the oropharynx into the esophagus to a depth sufficient for the tip to rest in the stomach. Adequate placement was confirmed by auscultation of stomach sounds during insufflation of air via syringe, followed by withdrawal of stomach contents through the central gastric access pathway within the device. The heat exchange unit was operated in patient control mode, in which the Medi-Therm III machine automatically regulates the patient’s temperature to the selected set point. The machine constantly compares actual patient temperature with the set point value, and automatically adjusts the water temperature so that the desired patient temperature is achieved.

### Measurements

**Figure 2:**
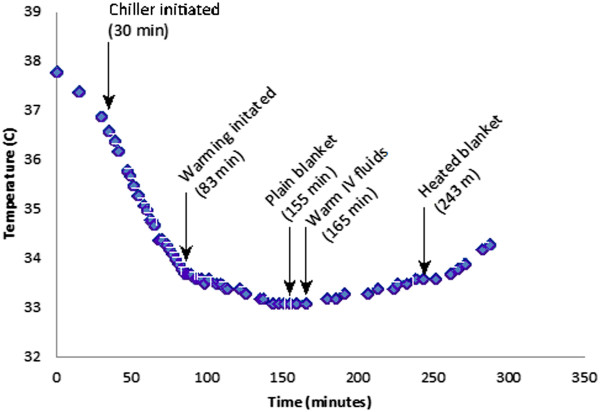
**Temperature over time for Swine #1.**

Temperature was recorded with a YSI-400 series compatible rectal temperature probe which was connected to the input patient probe connector on the external heat exchange unit, providing a continuous digital display of each subject’s temperature. In the first swine, we attempted to determine a maximum rate of temperature decrease from baseline to a goal temperature (4°C below baseline) by switching the external chiller to a maximum cooling mode 30 minutes after beginning the experiment. However, a baseline decrease in temperature was observed even prior to initiation of cooling with the experimental device (Figure 2). This outcome was attributed to cold laboratory conditions, including a fixed air vent blowing cold air into the laboratory, a cold metal laboratory table surface, and an uncovered animal. Additional measures, as discussed in the Results section below, where then undertaken to compensate for these conditions.

## Results

Although the first subject demonstrated a maximum temperature decrease of 3.5°C/hr, passive cooling theoretically may have contributed up to 1.8°C/hr of this rate. Likewise, due to passive losses in the cold laboratory environment, upon switching the chiller to warming mode at 83 minutes, additional decreases in temperature were seen (albeit at a much slower rate). In order to achieve full reversal of temperature decrease, the subject was covered at 155 minutes, and warm IV fluids were substituted for the room temperature fluids initially administered at 165 minutes into the protocol. These measures were sufficient to counteract the ongoing passive heat losses and resulted in a demonstrable increase in temperature (at a rate of 0.34°C/hr). Finally, at 243 minutes into the protocol, an external heated water blanket was applied and set to 39°C, resulting in further increase in the rate of temperature gain (to a rate of 0.95°C/hr).

**Figure 3:**
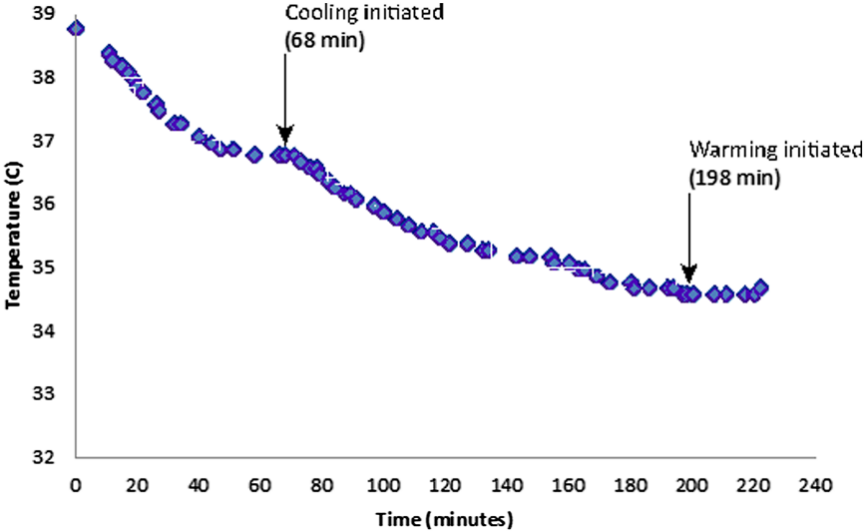
**Temperature over time for Swine #2.**

**Figure 4:**
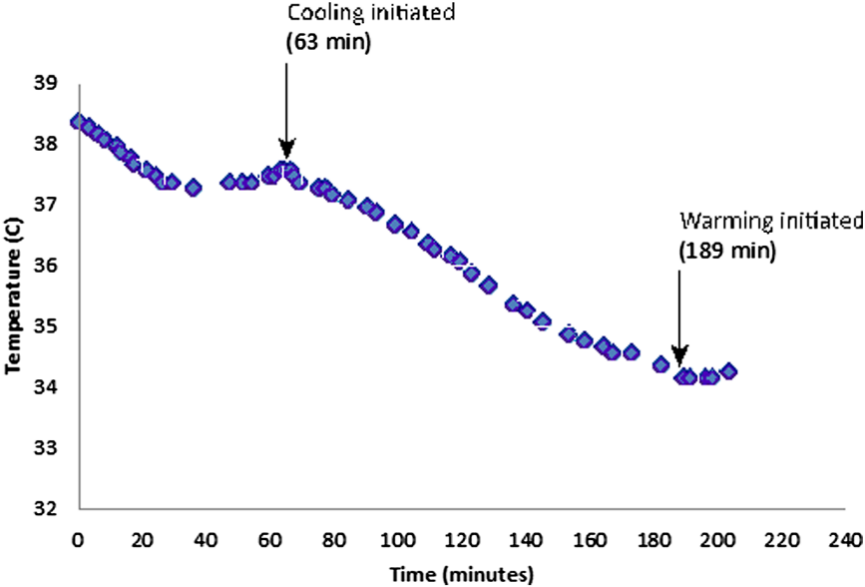
**Temperature over time for Swine #3.**

To compensate for the substantial ambient heat losses encountered in the laboratory, body temperature was stabilized prior to initiating cooling in the subsequent 2 swine. Stabilization methods included passive warming in the form of blankets to provide skin surface coverage, and using warmed IV fluids throughout the protocol. Once temperature was stabilized (68 minutes into the protocol for swine #2, shown in Figure 3, and 63 minutes into the protocol for swine #3, shown in Figure 4), the external heat exchanger was set to cooling mode, and temperature was monitored until goal temperature of 4°C below baseline was attained, at which point the external heat exchanger was set to a warming mode. Swine #2 and #3 exhibited temperature decreases of 1.7°C/hr. Cooling was successfully stopped and reversed at the end of the protocol in both cases through the use of the heat exchanger set to warming mode (although technician availability limited the length of time available for monitoring and precluded a complete return to baseline temperature prior to sacrifice). Warming rates achieved were 0.25°C/hr and 0.29°C/hr, respectively.

Histopathology of the esophagus of all three animals, performed after necropsy by a certified veterinary pathologist immediately after device removal using standard microscopic evaluation of thin sections of tissues, revealed normal tissue without evidence of injury.

## Discussion

Although a variety of approaches currently exist for controlling a patient’s temperature, many have inherent limitations. Surface devices are cumbersome, uncomfortable to surrounding operative staff, and of limited effectiveness when removed to gain access for surgical procedures. Intravascular catheters require sterile placement into the vasculature and the consequent risk of blood clots or infections. Controlling a patient’s temperature through the gastrointestinal tract has been accomplished with varying degrees of efficacy, both for cooling [39–42] and for warming [43–46] using various techniques and approaches. Using a free installation of liquid (water, or normal saline) that is either cooled with ice or warmed as appropriate for the intended effect has been the most common method of temperature control through the gastrointestinal tract, and is utilized as a mechanism of inducing therapeutic hypothermia in various hospitals [47, 48]. Warming efforts in the past have been less successful [46], and interest in this approach to temperature management specifically for warming appears to have diminished after publication of a letter which our research group recently found to contain an incorrect mathematical analysis [49]. In the device evaluated in this report, liquid is contained in a closed circuit system, avoiding the risks and potential complications of instilling free liquid into the gastrointestinal system. Additionally, the device tested in this investigation likely leverages more advanced materials, design specifications, and tolerances than were available in earlier investigations, allowing for greater performance and efficiency than has been achieved in earlier attempts to induce temperature change through the gastrointestinal tract.

We encountered greater ambient heat loss than initially expected. Cold laboratory conditions, combined with the small size of the swine, caused an unexpectedly high rate of heat loss, prompting the use of passive (blankets) as well as active (warmed IV fluids) warming methods to compensate. This permitted a more accurate determination of the cooling capability of the device, but limits the ability to make definitive conclusions regarding the maximum potential warming rates. It is likely that this device alone would not be sufficient to counter the threat of perioperative hypothermia in the most extreme cases (prolonged surgeries with extensive thoraco-abdominal exposure, for example); however, this esophageal heat transfer approach may be useful as an adjunct to these cases, and in less intense heat-loss situations, may be sufficient as the sole modality to avoid inadvertent perioperative hypothermia.

Placement of a device into the esophagus for the purpose of controlling patient temperature is more invasive than the use of external surface heat transfer devices; however, as compared with intravascular catheter devices used for this purpose, an esophageal approach can be considered less invasive. Additionally, an esophageal device requires no sterility for placement, and many patients undergoing temperature management, such as in the intensive care unit or operating room, receive a standard nasogastric or orogastric tube anyway.

Limitations of this investigation include the small sample size utilized. Although a larger sample would provide more refined data, the current data generally support the hypothesis that clinically significant modulations in temperature are possible in a pediatric model, and suggest that this may hold true in pediatric patients as well. Isoflurane may have contributed to systemic vasodilation and consequent increase in heat loss to the environment, particularly at the upper ranges of concentration utilized. We only measured temperature rectally, and rectal temperatures are known to lag those obtained from either bladder or intravascular means. Nevertheless, the expected differences in rates of temperature change are unlikely to affect our overall conclusions on the ability of this approach to have the intended effect on body temperature.

## Conclusions

In conclusion, we found that an esophageal heat transfer device successfully modulated the temperature of a pediatric swine model. This approach to temperature modulation may offer a useful new modality to control temperature in conditions warranting temperature management, such as maintenance of normothermia during surgery, induction of hypothermia for a growing number of indications (cardiac arrest, hypoxic ischemic encephalopathy, traumatic brain or spinal cord injury, stroke, etc.), fever control, or in the treatment of malignant hyperthermia.
